# Supplementary material for: Lime-based supplement reduces calcium oxalate stone recurrence: A multicenter randomized controlled trial
Source: PLoS One. 2025 Dec 5;20(12):e0336892. doi: 10.1371/journal.pone.0336892 (PMC12680185; doi:10.1371/journal.pone.0336892)

7. Study protocol

7.1 Study Population

The study population comprises patients diagnosed with kidney stones in Thailand. Participants will be recruited and enrolled from various hospitals across different regions of the country. The clinical trial will be conducted through a multicenter research network comprising eight participating sites.

7.2 Inclusion Criteria

- Ages 18-70 years old, both sexes
- Solitary stone of any size, in any locations in the kidney (except ureters, lower urinary stones)
- Radiology of any modality proving the existence of the stone (ultrasound, computed tomography, intravenous pyelogram, kidney-ureter-bladder (KUB) x-ray)
- For clinical trial, patients' kidneys must be stone-free evaluated at 1 month after operation by KUB x-ray or ultrasound.

7.3 Exclusion Criteria

- Ages < 18 years old , Age > 75 years old
- Infection or magnesium ammonium phosphate stone
- Pregnant females
- Male patients treated for with benign prostate hyperplasia (BPH) (ongoing medical treatment or surgical intervention within 6 months)
- Positive urine culture
- Any malignancies
- Chronic recurrent urinary infections (prostate, cystitis, vaginosis/vaginitis)
- Gross hematuria
- Autoimmune disease that may affect renal function (e.g. Systemic lupus erythematosus)
- Renal dysfunction or its common causes: diabetes, uncontrolled hypertension (with concurrent microalbuminuria) (diastolic BP > 90 mmHg), glomerulonephritis,
- Renal transplant
- Genetic stone disease (e.g., cystine stones, xanthinuria)
- Medullary sponge kidney, or other renal anatomic anomalies such as horseshoe kidney
- GI disorders: Inflammatory bowel disease, short bowel
- Hypercalcemic disorders (hyperparathyroidism, sarcoidosis, Paget's disease)
- Renal tubular acidosis
- Immunocompromised patients e.g. HIV (indinavir stones)
- Bleeding disorders
- Unable to provide informed consent
- Anyone in the opinion of the investigator who would be inappropriate

7.4 Discontinuation Criteria

7.4.1 Criteria for Voluntary Withdrawal by Participants

Participants enrolled in this study have the right to withdraw from the trial at any time without the need to provide a reason. Such withdrawal will not affect the participant's standard medical care in any way. The research team will make every effort to conduct the study with the

utmost care to avoid any harm to participants. In the event of any adverse events or injuries directly related to the study, the research team will ensure fair compensation and appropriate care.

7.4.2 Criteria for Termination of the Entire Study

The principal criterion for early termination of the study is the occurrence of serious adverse events associated with the investigational product (LPR). Such events will be evaluated by the treating physician and/or the Institutional Review Board (IRB) or Ethics Committee overseeing the study.

7.5 Study Procedures, Research Control, Data Collection, and Statistical Analysis

7.5.1 Sample Size Calculation

Based on historical data, the recurrence rate of kidney stone formation is approximately 40% over a period of 2.5 years (30 months). This study aims to evaluate the reduction in recurrence rates between participants receiving the Lime Powder Regimen (LPR) and those receiving placebo. The anticipated effect size is a 40% reduction in recurrence risk, corresponding to a hazard ratio (HR) of 0.6. This effect is also interpretable as an extension in time to recurrence from 30 months to 50 months.

Sample size was calculated using the Cox Proportional-Hazards Regression model according to the method outlined by Hsieh and Lavori, Schoenfeld, and Chow et al. The following parameters were applied in the calculation: Power (1–β): 90% (β = 0.10), Significance level: α = 0.05 (two-sided), Hazard ratio (HR): 0.6 and Coefficient of determination (R^2) of covariates in the model: 0.05

$$N = \frac{(Z_{\alpha/2}+Z_{\alpha})^2}{b^2 p_1 p_2 d(1-R^2)} \quad \text{.......(1)}$$

When $Z_{\alpha/2}$ = 1.96 (corresponding to a two-sided type I error of α = 0.05)

Z_{α} = 1.282 (for statistical power of 90%)

b^2 = log(Hazard Ratio) = log(1 / 0.6) = 0.51082 ≈ 0.261

p_1 = Proportion of participants in the control group = 0.5

p_2 = Proportion of participants in the experimental group = 0.5

d = Event rate (recurrence of kidney stones) = 0.40

R^2 = Coefficient of determination for covariates = 0.05

By substituting these values into the sample size calculation formula (Equation 1), the number of participants required in each group can be determined.

$$N = \frac{(1.96 + 1.282)^2}{0.5108^2 x0.5x0.5x0.4x(1 - 0.05)}$$

The initially calculated sample size was 71 participants per group. To account for potential participant dropouts, the sample size was adjusted using the standard sample size inflation formula:

$$N_{adjusted} = \frac{1}{[1 - dr]}$$

Based on previous studies, the dropout rate (dr) for participants was estimated at 10%. After adjusting to this expected attrition, the sample size was increased to 78 participants per group. Thus, this Phase III clinical trial initially required a total of 156 patients with kidney stone disease, randomly allocated into two equal groups: Intervention group (Lime Powder Regimen): 78 patients and Control group (Placebo): 78 patients. However, due to the long-term follow-up period of 2 years, which may result in higher-than-expected participant attrition, the research team decided to increase the projected dropout rate to 15%. Based on this revised estimate, the adjusted

sample size was recalculated, and 90 participants in each group was desirable, and total participants are 180 persons.

7.5.2 Intervention: Lime Powder Regimen and Placebo Administration

Patients with kidney stone disease who had either no residual stones or residual stones smaller than 4 mm, assessed one-month post-surgery, were eligible for inclusion in the study.

1. Eligible participants were randomly assigned in a double-blind fashion to receive either the Lime Powder Regimen (LPR) or a placebo (lactose). Both interventions were administered as 5-gram powder sachets, sealed in light-resistant packaging. Participants were instructed to dissolve one sachet daily in 250–500 mL of clean drinking water and to consume the solution within 5 minutes, preferably before bedtime. Each sachet of the Lime Powder Regimen contained 63 mEq of citrate and 21 mEq of potassium. The placebo sachet consisted of slightly sweet-tasting lactose, without added flavor or aroma. Notably, LPR has a sour taste, which may potentially compromise the blinding process. However, this concern was mitigated by the following considerations: Participants were recruited from geographically diverse locations, minimizing the likelihood of communication or comparison between subjects; Each participant consistently received the same intervention throughout the study duration; Blinding integrity was preserved by avoiding the use of additional flavoring agents in the placebo, which could independently affect metabolic pathways associated with kidney stone formation or recurrence. According to regional urological guidelines, following surgical management of urolithiasis—including nephrolithiasis, ureterolithiasis, cystolithiasis, and urethrolithiasis—patients are not prescribed medications or supplements known to influence stone recurrence risk, apart from dietary and lifestyle counseling. Therefore, the research team is confident that any observed effects are attributable solely to the investigational intervention (Lime Powder Regimen or lactose placebo), without confounding by co-administered stone-modifying agents.

2. Study Duration and Follow-Up Schedule

This Phase III clinical trial involves a 24-month intervention period, during which enrolled patients are required to take the assigned study medication daily for two years. Participants will attend five scheduled clinical visits for follow-up assessments at baseline (Month 0), Month 6, Month 12, Month 18, and Month 24.

3. At each visit, the following evaluations will be conducted:

3.1 Renal Function Tests: Blood Urea Nitrogen (BUN) and Serum Creatinine will be measured at Months 0, 12, and 24 to assess kidney function.

3.2 Hepatic Function Test: Alanine aminotransferase (ALT) levels will be assessed at Months 0, 12, and 24 to monitor liver safety.

3.3 Urinalysis: Routine urinalysis, including protein quantity and type, will be performed at all five visits (Months 0, 6, 12, 18, and 24) to evaluate changes in urinary composition and detect any early signs of renal deterioration or stone recurrence.

3.4 Computed Tomography (CT) Scan: Non-contrast CT scans will be conducted at Months 0, 12, and 24 to assess the development of new kidney stones or recurrence of previous stones with higher sensitivity.

All assessments will be carried out in the morning, prior to physician evaluation. Fasting is not required for any of the tests.

About the biobanking for future analysis, with participant consent, blood and urine samples collected during study visits will be stored for future exploratory analyses of

biochemical markers. Sample storage will not exceed 10 years, and all future analyses will be conducted under appropriate ethical approvals.

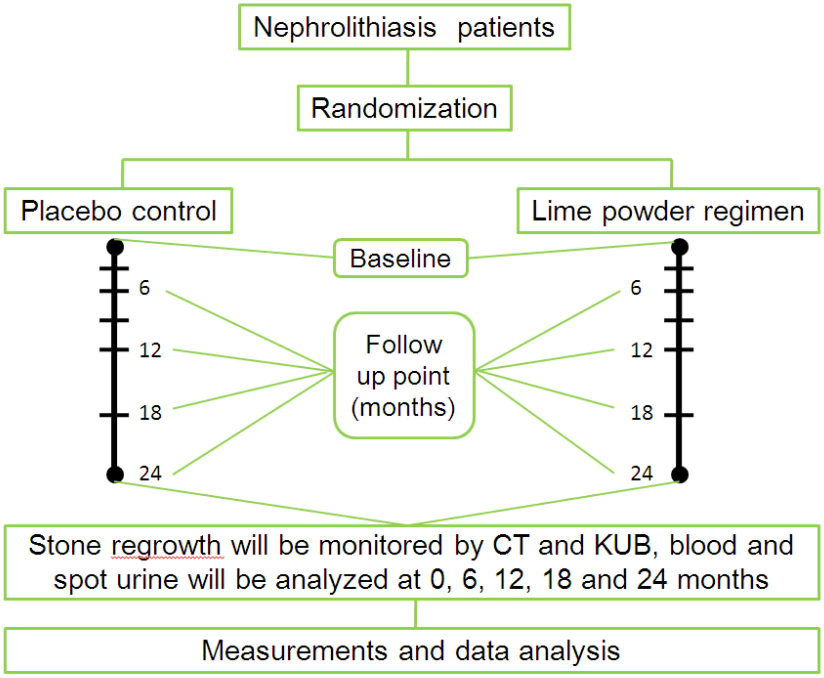

Supplement: S1 File — (PDF) [file pone.0336892.s001.pdf]
