# Supplementary material for: Lime-based supplement reduces calcium oxalate stone recurrence: A multicenter randomized controlled trial
Source: PLoS One. 2025 Dec 5;20(12):e0336892. doi: 10.1371/journal.pone.0336892 (PMC12680185; doi:10.1371/journal.pone.0336892)
Supplement: S2 File — This file contains additional supporting materials for the study, including Table S1 (metal concentration in lime product), Table S2 (baseline demographic data), Table S3 (nutritional value of 24-hour duplicated meals), Table S4 (summarization of secondary outcomes and interpretation), and Figure S (24-hour urinary parameters between groups). These materials support the findings presented in the main text. (DOCX) [file pone.0336892.s002.docx]

Table S1 Determination of heavy metal concentrations in LPR

| Chemical constituents | Amount (ppm) | ALDC-HM USP reference (ppm) | OPDE-DF USP reference (mg/day) | RDA  (mg/day) |
| --- | --- | --- | --- | --- |
| Metals  Arsenic (As) | 0.01 | 1.5 | 0.015 | - |
| Cadmium (Cd) | UD | 0.5 | 0.025 | - |
| Cobalt (Co) | UD | nd | 0.10 | - |
| Chromium (Cr) | UD | 25 | 0.15 | 0.0002-0.05 |
| Nickel (Ni) | 0.06 | 25 | nd | - |
| Lead (Pb) | 0.02 | 1 | 0.01 | - |
| Mercury (Hg) | 0.01 | - |  | - |
| Antimony | 0.01 | nd | 0.02 | - |
| Vanadium (V) | 0.01 | 25 | nd | - |

USP: United States Pharmacopoeia; ALDC-HM: Acceptable Limits for Daily Consumption of Heavy Metals; OPDE-DF: Oral Permitted Daily Exposure for Dosage Forms; RDA: Recommended Dietary Allowances; ppm: part per million; UD: undetectable; nd: no data

Table S2 Baseline demographic and clinical characteristics of participants between complete participants and drop-out

| Parameters | Complete | Dropped out | p-Value |
| --- | --- | --- | --- |
| Participants (persons) | 151 | 22 |  |
| Gender: Male (%) | 59.6 | 50.0 | 0.013 |
| Age (years, mean ± SD) | 50.35±7.94 | 48.33±7.44 | NS |
| Residential area |  |  |  |
| Northeast region (%) | 73.5 | 88.9 | NS |
| North region (%) | 11.3 | 5.6 | NS |
| Central region (%) | 10.6 | 5.6 | NS |
| East region (%) | 4.6 | 0 | NS |
| Type of stone  Calcium oxalate  Mixed calcium stone | 89.4%  10.6% | 86.4%  13.6% | NS  NS |
| Number of stones present at diagnosis | | | |
| 1 stone  >1 stone | 78.8%  22.2% | 77.3%  22.% | NS  NS |

Table S3 Nutritional value of 24-hour duplicated meals from 11 LPR participants and 11 Placebo participants

| Nutrients | LPR | Placebo | p-value |
| --- | --- | --- | --- |
| Energy (Kcal/day) | 1,205.6+311.8 | 1,125.0+144.1 | 0.949 |
| Carbohydrates (g/day) | 178.2+42.4 | 174.5+15.8 | 0.699 |
| Lipid (g.day) | 28.3+14.6 | 24.5u+9.7 | 0.480 |
| Protein (g/day) | 59.5+20.8 | 51.7+13.6 | 0.317 |
| Calcium (g/day) | 459.7+357.1 | 293.1+186.3 | 0.478 |
| Iron (mg/day) | 9.8+2.8 | 8.9+2.3 | 0.438 |
| Sodium (Mg/day) | 3,941.1+2,725.3 | 2,840.9+903.6 | 0.438 |
| Phosphorus (mg/day) | 577.0+292.2 | 489.5+137.6 | 0.797 |
| Potassium (mg/day) | 1,215.0+473.4 | 1,054.1+228.2 | 0.327 |
| Magnesium (mg/day) | 36.46+19.52 | 33.74+14.17 | 0.720 |
| Vitamin A (μg/day) | 252.4+249.4 | 480.1+972.3 | 0.652 |
| Vitamin B1 (mg/day) | 1.16+1.54 | 1.00+0.81 | 0.562 |
| Vitamin B2 (mg/day) | 1.04+0.64 | 0.93+0,44 | 0.562 |
| Vitamin B3 (mg/day) | 13.09+4.71 | 11.85+2.35 | 0.466 |
| Vitamin B6 (mg/day) | 0.22+0.14 | 0.30+0.24 | 0.519 |
| Vitamin B12 (μg/day) | 0.62+0.68 | 1.30+2.19 | 0.652 |
| Vitamin C (mg/day) | 92.60+60.48 | 59.73+23.58 | 0.300 |
| Vitamin E (mg/day) | 10.91+23.02 | 3.17+7.03 | 0.847 |
| Fiber (g/day) | 11.15+6.19 | 9.30+3.06 | 0.390 |

Table S4 Summarization of secondary outcomes and interpretation

| Outcome | LPR (Mean$\pm$SD) | Placebo (Mean$\pm$SD) | 95% CI | *p*-value | Interpretation |
| --- | --- | --- | --- | --- | --- |
| Urinary protein excretion (g/day) | 202.4$\pm$167.3 | 304.5$\pm$242.8 | 3.868 to 200.3 | 0.042 | Reduced in LPR |
| Urinary IL-8 (pg/mL) | 83.9$\pm94.8$ | 197.8$\pm140.4$ | 57.45 to 170.4 | <0.001 | Reduced in LPR |
| Serum creatinine (mg/dL) | 0.88$\pm0.$21 | 0.99$\pm0.24$ | -0.64 to 0.13 | 0.490 | No difference  (No renal toxicity) |
| Serum BUN (mg/dL) | 12.79$\pm$4.13 | 13.37$\pm$3.72 | -1.07 to 2.22 | 0.489 | No difference  (No renal toxicity) |
| eGFR (mL/min/1.37m^2^) | 91.72$\pm$19.27 | 82.18$\pm18.43$ | -8.92 to 4.60 | 0.529 | Stable in both  (No renal toxicity) |
| ALT (U/L) | 24.96$\pm$13.18 | 26.29$\pm$13.97 | -4.46 to 7.13 | 0.648 | No difference  (No hepatic toxicity) |


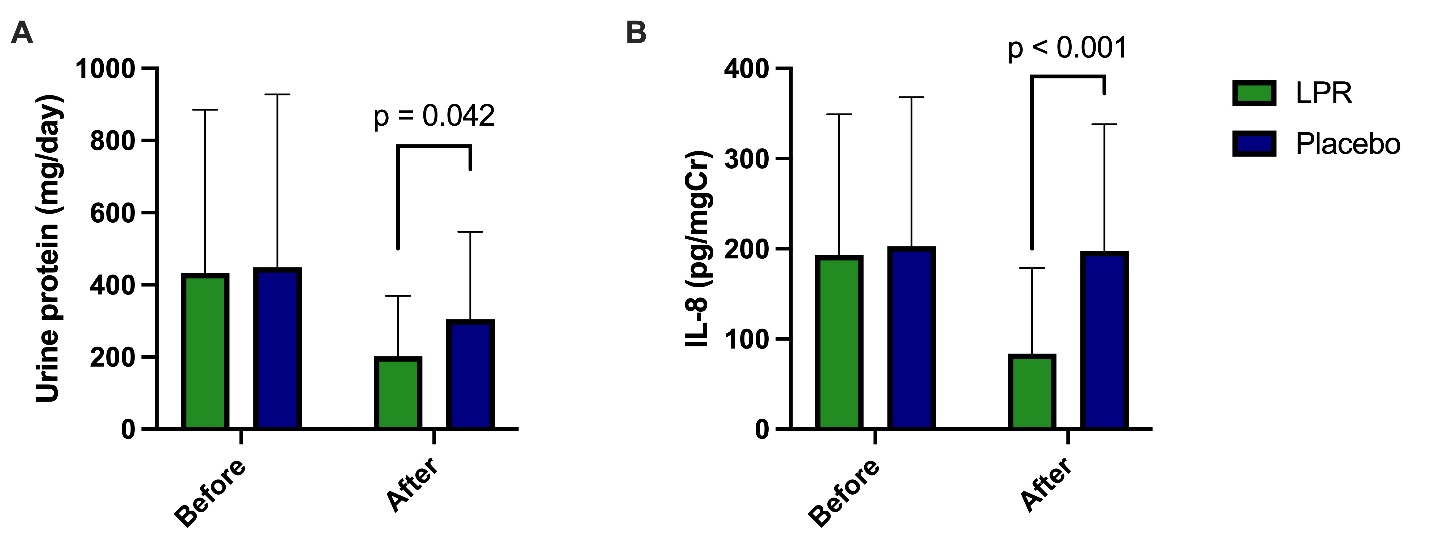


Figure S1: Comparison of 24-hour urinary parameters between the placebo and LPR groups at baseline and at 24-month follow-up. (A) Urinary protein excretion was significantly reduced in the LPR group compared with the placebo group. (B) Urinary IL-8 levels were also reduced in the LPR group relative to placebo. Analyses were performed using unpaired t-tests, and error bars indicate standard deviations.

Additional information:

Sample size calculation

Followed by the “Medical management to prevent recurrent nephrolithiasis in adults: a systematic review for an American College of Physicians Clinical Guideline” Ann Intern Med. 2013 Apr 2;158(7):535-43.

Urolithiasis patients received citrate treatment had the relative risk of 0.25 (0.14-0.44) for stone recurrence

Using the equation of


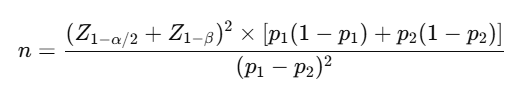


Alpha = 0.05, and power = 80%

- Required sample size per group: 72 participants
- Total sample size (both groups): 145 participants
