# Supplementary material for: Lime-based supplement reduces calcium oxalate stone recurrence: A multicenter randomized controlled trial
Source: PLoS One. 2025 Dec 5;20(12):e0336892. doi: 10.1371/journal.pone.0336892 (PMC12680185; doi:10.1371/journal.pone.0336892)
Supplement: Attachment — Submitted filename: Comments_SJ.docx [file pone.0336892.s003.docx]

This is a well-designed study on an attractive and important issue. The novelty lies in linking a dietary intervention with inflammation-related mechanisms in the context of recurrent calcium oxalate urolithiasis. The trial addresses a clinically relevant gap and is well presented.

**Abstract**

The abstract is generally clear and well written.

Suggestion: In the results section, report the absolute amount of reduction in urinary IL-8 and protein excretion, in addition to p-values, to better convey clinical importance.

**Introduction**

- **References:** Some cited references are dated.
  - **Lines 52–54:** References 1–3 (incidence of urolithiasis) should be updated with more recent epidemiological data.
  - **Line 56:** References 4–6 supporting urinary metabolic abnormalities are dated and population-limited. Specifically, reference #6 appears less relevant, as it focuses on urinary citrate excretion and therapeutic interventions rather than directly linking urinary metabolic abnormalities to stone risk. In addition, the cited studies are relatively dated and limited to Thai populations, which may restrict their generalizability. Replace with more recent, high-quality reviews.
  - **Line 59:** The current references (6, 7) cited for the statement that “potassium citrate therapy is the standard pharmacological intervention to prevent recurrence” are limited, particularly reference #7, which is based on a small mechanistic study.I would also caution against relying exclusively on references (6, 7), as both are authored by the same research team and may introduce unnecessary self-citation bias. Given that the claim relates to standard pharmacological intervention, it would be more appropriate to cite authoritative clinical practice guidelines or comprehensive consensus statements. Like: European Association of Urology (EAU) Guidelines on Urolithiasis which recommend potassium citrate as first-line pharmacological therapy for recurrent calcium stone formers with hypocitraturia. or (AUA) Guideline on Medical Management of Kidney Stones
  - **Line 60:** References 8 and 9 (1985 and 2005) are outdated and do not reflect current pharmacological practice. Since then, potassium citrate formulations have evolved—for example, coated and extended-release preparations designed to reduce gastrointestinal side effects. Therefore, it would be important to replace or supplement these older citations with more recent, guideline-based and systematic review evidence. Stronger references should be provided to support adverse effects and adherence: recent studies highlight that, despite improved formulations, gastrointestinal intolerance and poor adherence remain major limitations, compounded by the high cost of long-term therapy, particularly in low-resource settings.
  - **Lines 63, 60:** Ensure periods are consistently placed outside reference parentheses.
  - **Line 68:** The claim that renal inflammation contributes to stone pathogenesis requires citation of recent studies.
  - **Line 72:** Please provide the **citrate content** of the lime-based regimen here.
  - **Line 76:** Expand on how IL-8 and urinary protein can serve as biomarkers of treatment response.

**Methods**

- **Line 85:** Age eligibility is inconsistent: the protocol exclusion criteria mention ≤75 years, while the text states ≤70 years. Please clarify.
- **Line 89:** The eligibility criterion in the Supplementary Material (residual stones ≤4 mm at 1 month post-surgery) is clearer than in the main text. Harmonize these.
- **Line 91:** Sample size assumptions in supplementary material: In the sample size calculation section, you state that the historical recurrence rate is ~40% over 2.5 years and that the study anticipates a 40% reduction in recurrence risk (HR = 0.6), with calculations based on methods by Hsieh and Lavori, Schoenfeld, and Chow et al. However, no supporting references are provided for either the historical recurrence rate assumption or the anticipated effect size.
- **Line 94:** Please include in the main text (not only in Supplementary Material) that each LPR sachet contained **63 mEq citrate and 21 mEq potassium**. Compare this dosage with standard potassium citrate prescriptions (30–60 mEq/day). Note that this dose is at the *upper end* of recommended citrate therapy, but with less potassium. Providing this comparison would help readers better interpret the clinical relevance of the intervention relative to established therapies.
- **Placebo composition:** Please report the exact lactose content per placebo sachet and discuss whether the amount falls below the usual tolerance threshold (≈12 g). Also clarify whether screening for lactose intolerance was considered, given its relatively high prevalence in Asian populations.
- **Line 105:** Dose of 7.75 g LPR — please provide rationale (e.g., based on prior data, pharmacokinetics, or pilot testing). State explicitly how much *bioavailable citrate* this dose provides in mEq/day.
- **Line 115:** The 24-hour urine protocol is unclear. Was urine collected only once at study end and baseline? Were duplicate collections performed to improve accuracy? Why was urinary citrate and other stone-related metabolites not reported?
- **Line 122:** The safety monitoring process is clearly described; however, the handling of clinical events requiring intervention during follow-up is unclear. Please clarify: Management of recurrence requiring intervention: If, during the 2-year follow-up, a participant developed a stone of sufficient size or symptoms to warrant surgical removal or another intervention, how was this managed within the trial? Were such patients excluded from further follow-up analyses? Or were they retained and analyzed according to the intention-to-treat principle?

**Results (and Tables/Figures)**

- **Line 147:** The allocation of 98 participants to the intervention arm and 75 to the placebo arm suggests an unequal randomization ratio. Could the authors clarify How was the sample size determined, and did this imbalance arise by chance due to the randomization process? Loss of participants after randomization, allocation errors, or differential withdrawal before intervention).
- **Line 149**: The phrase “number of stone and recurrence rate” is confusing — “stonr number” already mentioned in the sentence and “recurrence rate” at baseline is ambiguous since recurrence is typically a follow-up outcome.
- **Baseline data (Table 1):**
  - Consider adding stone size as a baseline variable.
  - Note that males were underrepresented compared to expected prevalence. This is noteworthy, as epidemiological evidence consistently shows that the prevalence and incidence of urolithiasis are higher in men than in women. Could it indicate selection bias in enrollment? Could the predominance of female participants influence the generalizability of the findings, given known sex-related differences in stone risk and recurrence?
  - Type of stone: In the Methods section, there is no description of how stone types were determined.
- **Line 152**: I recommend comparing the baseline characteristics of participants who were lost to follow-up with those who completed the study. This would help assess whether attrition introduced potential bias.
- **Line 166:** The section on Detection of LPR contamination appears redundant, as similar information has already been provided under LPR manufacturing, quality control and supplementation. To improve conciseness, you could consolidate these details into a single section. For example, heavy metal and pathogen testing results could be summarized once under quality control, with Table S1 referenced there if needed, rather than repeating them in multiple places.
- **Line 173:** The phrase “stone recurrent rate” is not standard; the appropriate term would be “stone recurrence rate”. I recommend revising the wording accordingly.
- The manuscript states that stone recurrence was monitored by KUB radiography (6 and 18 months) and CT (12 and 24 months). However, this is more a description of the imaging schedule rather than a clear definition of recurrence. For clarity, please specify: Was recurrence defined as the appearance of a new stone, the growth of a pre-existing stone, or the need for clinical intervention? Additionally, please ensure that “recurrence rate” is defined consistently in the manuscript. Clear and consistent terminology will improve readability and scientific precision.
- **Line 198:** A short comment in the Discussion on the clinical relevance of decreased urinary protein and IL-8 levels in relation to stone recurrence would strengthen interpretation.
- **Line 202:** Please clarify whether the reported changes in urinary protein and IL-8 are (a) within-group changes from baseline or (b) between-group differences versus placebo. For efficacy inference in an RCT, the primary analysis should compare LPR vs placebo, adjusting for baseline values. report adjusted between-group differences (or ratios if log-scaled) with 95% CIs and P-values; avoid relying on within-group significance alone.
- **Line 203:** A paired t-test is appropriate for within-group baseline vs. follow-up comparisons, but not for comparing intervention and placebo groups in an RCT. For efficacy inference, the main analysis should be a between-group comparison, ideally adjusting for baseline values

**Discussion**

- **Line 208**: The introduction and methods do not provide any information on the phytochemical content of the lime-based supplement. However, the discussion opens by emphasizing its “phytochemical-rich” properties and links the efficacy to this aspect. This could be misleading, as no phytochemical analyses were conducted or reported in the trial. The observed effects are more plausibly attributable to citrate or other components that were not specifically tested. I recommend revising the discussion to focus on conclusions that can be directly supported by the RCT data (e.g., changes in recurrence, urinary parameters, safety outcomes), while avoiding speculative statements about phytochemicals unless supported by measured evidence. If phytochemicals are to be discussed, they should be presented clearly as a potential mechanism requiring further study rather than as a proven explanation.
- **Line 210:** Please discuss **cost-effectiveness** of LPR compared to standard potassium citrate therapy. Cost-effectiveness is a key factor in long-term adherence and clinical adoption, particularly in low-resource settings where stone recurrence burden is high. Addressing whether LPR offers an economic advantage compared with existing pharmacological options would provide valuable context for interpreting its potential role in clinical practice.
- **Line 225**: Here In the discussion, you state that urinary protein reflects both glomerular and tubular damage associated with crystalluria and inflammation. However, this important claim is currently supported by only one small, self-cited study. For a statement of this scope, I strongly recommend citing multiple, higher-quality sources. In particular, please incorporate recent reviews and large observational studies that: Document crystal-induced tubular injury and inflammatory pathways in kidney stone disease. Summarize evidence for tubular injury biomarkers (e.g., KIM-1, NGAL) and their relevance to stone pathophysiology. Highlight epidemiologic data linking albuminuria/proteinuria with increased risk of nephrolithiasis. Expanding the reference base in this way will strengthen the scientific foundation of your argument and avoid overreliance on a single study from your own group.
- **Line 230**: Flavonoids and related polyphenols can reduce oxidative stress/inflammation, modulate crystal growth/adhesion, and influence crystal–cell interactions in vitro/animal models; clinical translation remains limited. If mentioned, please frame as hypothesis and cite reviews rather than implying causality.
  While citrus-derived phytochemicals have been shown in preclinical models to reduce oxidative stress, modulate crystal growth/adhesion, and attenuate inflammatory signaling, including pathways related to IL-8, these mechanisms were not directly evaluated in our trial. The observed clinical benefit is more parsimoniously explained by alkali/citrate effects, consistent with prior citrus-juice and citrate literature; phytochemical mechanisms should be viewed as hypotheses for future targeted studies.
- **Line 251**: In the conclusion, the manuscript emphasizes “phytochemical-based therapies” as a potential strategy for secondary prevention. However, this conclusion is not supported by the data presented. The trial did not measure or report the phytochemical content of the intervention, nor did it test mechanistic pathways to link phytochemicals with outcomes. The observed benefits are more directly attributable to the citrate content of the supplement and related metabolic effects.
- **Line 260**: It is a valuable consideration that dietary intake was assessed across groups, as diet can influence urinary chemistry and stone recurrence. I suggest repeated dietary recalls for this matter.

**Conclusion**

- I recommend revising the conclusion to reflect findings that are directly supported by this RCT—namely, the observed reduction in stone recurrence and urinary/biochemical changes with LPR. Phytochemicals may be mentioned only as a hypothesis-generating area for future research, rather than as evidence-based therapies suitable for guideline integration at this stage.
- The reported 75.6% reduction in recurrence should be clarified as relative vs absolute risk, and effect sizes presented with confidence intervals.
- Statements about uric acid and calcium phosphate stones are speculative. Please restrict conclusions to **calcium-based stones**, which were studied.
  - The trial enrolled patients with calcium-based stones (predominantly calcium oxalate). Therefore, claims that LPR may also benefit patients with uric acid stones or be ineffective for calcium phosphate stones are speculative and not supported by your data. I recommend revising the conclusion to clearly state that the findings apply to calcium-based stone formers only, and note that efficacy in other stone types requires further investigation.
- The mechanisms listed (citrat­uria, alkalinization, antioxidant/anti-inflammatory effects from citrus flavonoids) should be revised. None of these were directly demonstrated in this trial. The proposed role of flavonoids is speculative, as no phytochemical analyses or mechanistic assessments were conducted. Please frame flavonoid-related mechanisms as hypotheses for future research rather than established findings. While it is appropriate to discuss these possibilities in the Discussion, they should not be included in the Conclusion. Revise to highlight **evidence-based findings**: reduced recurrence and improved urinary parameters in calcium-based stone formers.
- Emphasize that safety was observed over 2 years, but long-term tolerability remains to be evaluated. Maintain caution regarding potassium use in advanced CKD, with reference to guidelines.
