# Supplementary material for: Lime-based supplement reduces calcium oxalate stone recurrence: A multicenter randomized controlled trial
Source: PLoS One. 2025 Dec 5;20(12):e0336892. doi: 10.1371/journal.pone.0336892 (PMC12680185; doi:10.1371/journal.pone.0336892)
Supplement: Attachment — Submitted filename: renamed_1f037.docx [file pone.0336892.s005.docx]

Response to reviewers’ comments

| Comment | Correction |
| --- | --- |
| **Reviewer 1** | |
| **Introduction**  **References:** Some cited references are dated.  **Lines 52–54:** References 1–3 (incidence of urolithiasis) should be updated with more recent epidemiological data.  **Line 56:** References 4–6 supporting urinary metabolic abnormalities are dated and population-limited. Specifically, reference #6 appears less relevant, as it focuses on urinary citrate excretion and therapeutic interventions rather than directly linking urinary metabolic abnormalities to stone risk. In addition, the cited studies are relatively dated and limited to Thai populations, which may restrict their generalizability. Replace with more recent, high-quality reviews.  **Line 59:** The current references (6, 7) cited for the statement that “potassium citrate therapy is the standard pharmacological intervention to prevent recurrence” are limited, particularly reference #7, which is based on a small mechanistic study.I would also caution against relying exclusively on references (6, 7), as both are authored by the same research team and may introduce unnecessary self-citation bias. Given that the claim relates to standard pharmacological intervention, it would be more appropriate to cite authoritative clinical practice guidelines or comprehensive consensus statements. Like: European Association of Urology (EAU) Guidelines on Urolithiasis which recommend potassium citrate as first-line pharmacological therapy for recurrent calcium stone formers with hypocitraturia. or (AUA) Guideline on Medical Management of Kidney Stones  **Line 60:** References 8 and 9 (1985 and 2005) are outdated and do not reflect current pharmacological practice. Since then, potassium citrate formulations have evolved—for example, coated and extended-release preparations designed to reduce gastrointestinal side effects. Therefore, it would be important to replace or supplement these older citations with more recent, guideline-based and systematic review evidence. Stronger references should be provided to support adverse effects and adherence: recent studies highlight that, despite improved formulations, gastrointestinal intolerance and poor adherence remain major limitations, compounded by the high cost of long-term therapy, particularly in low-resource settings.  **Lines 63, 60:** Ensure periods are consistently placed outside reference parentheses.  **Line 68:** The claim that renal inflammation contributes to stone pathogenesis requires citation of recent studies.  **Line 72:** Please provide the **citrate content** of the lime-based regimen here.  **Line 76:** Expand on how IL-8 and urinary protein can serve as biomarkers of treatment response. | - We updated the reference to newer ones (reference no.1 to 3) as well as the corresponding statements in yellow highlights  - We updated the references to newer ones (Ref no 4-6) as your suggestion  -We updated the reference to the newer ones (Ref no.7-8) from EAU guideline and Nature review.  - We updated the references no. 9-10, however, even these manuscripts commented about gastrointestinal adverse effect of potassium citrate, but the trial performed in cystinuria and pediatric renal tubular acidosis, no urolithiasis. The latter is prolonged release of potassium citrate and potassium bicarbonate. Full stop is also corrected.  - We corrected the periods, and *italic* of *C. aurantifolia*  - We added references related to renal inflammation and oxidation-induced calcium oxalate stone formation (Ref no. 14-15).  - We added “*Each sachet of LPR contained 7.75 g of supplement, providing a citrate dose comparable to standard alkali therapy but with a reduced potassium content*.” in Methods section, LINE 104-109  - For proper arrangement, we added “*The observed clinical benefits are supported by mechanistic plausibility. Crystal-induced epithelial injury and micro-obstruction in stone disease promote local inflammation and cytokine release, including IL-8, thereby sustaining oxidative stress and facilitating crystal retention. Elevated urinary protein similarly reflects obstruction- or injury-related disruption of glomerular and tubular integrity. Reductions in IL-8 and proteinuria following LPR supplementation suggest attenuation of renal inflammation and restoration of barrier function, consistent with recovery of renal health (20). Prior recommendations from the Experts in Stone Disease (ESD) Conference also emphasize the utility of proteinuria monitoring in high-risk stone formers, underscoring the clinical relevance of these markers (21).”* in Discussion part, LINE 233-240 |
| **Methods**  **Line 85:** Age eligibility is inconsistent: the protocol exclusion criteria mention ≤75 years, while the text states ≤70 years. Please clarify.  **Line 89:** The eligibility criterion in the Supplementary Material (residual stones ≤4 mm at 1 month post-surgery) is clearer than in the main text. Harmonize these.  **Line 91:** Sample size assumptions in supplementary material: In the sample size calculation section, you state that the historical recurrence rate is ~40% over 2.5 years and that the study anticipates a 40% reduction in recurrence risk (HR = 0.6), with calculations based on methods by Hsieh and Lavori, Schoenfeld, and Chow et al. However, no supporting references are provided for either the historical recurrence rate assumption or the anticipated effect size.  **Line 94:** Please include in the main text (not only in Supplementary Material) that each LPR sachet contained **63 mEq citrate and 21 mEq potassium**. Compare this dosage with standard potassium citrate prescriptions (30–60 mEq/day). Note that this dose is at the *upper end* of recommended citrate therapy, but with less potassium. Providing this comparison would help readers better interpret the clinical relevance of the intervention relative to established therapies.  **Placebo composition:** Please report the exact lactose content per placebo sachet and discuss whether the amount falls below the usual tolerance threshold (≈12 g). Also clarify whether screening for lactose intolerance was considered, given its relatively high prevalence in Asian populations.  **Line 105:** Dose of 7.75 g LPR — please provide rationale (e.g., based on prior data, pharmacokinetics, or pilot testing). State explicitly how much *bioavailable citrate* this dose provides in mEq/day.  **Line 115:** The 24-hour urine protocol is unclear. Was urine collected only once at study end and baseline? Were duplicate collections performed to improve accuracy? Why was urinary citrate and other stone-related metabolites not reported?  **Line 122:** The safety monitoring process is clearly described; however, the handling of clinical events requiring intervention during follow-up is unclear. Please clarify: Management of recurrence requiring intervention: If, during the 2-year follow-up, a participant developed a stone of sufficient size or symptoms to warrant surgical removal or another intervention, how was this managed within the trial? Were such patients excluded from further follow-up analyses? Or were they retained and analyzed according to the intention-to-treat principle? | - We corrected the text in LINE 85 to “*18-75 years*”, following the protocol. Thank you for your suggestion.  - We corrected the statement into “*had residual stones ≤4 mm at 1-month post-surgery evaluated by KUB x-ray or ultrasound*.” LINE 88-89  - We appreciate your comment, but we could not find the information regarding your comment. For sample calculation we used “Medical management to prevent recurrent nephrolithiasis in adults: a systematic review for an American College of Physicians Clinical Guideline” Ann Intern Med. 2013 Apr 2;158(7):535-43. As mentioned in Supplementary. We added “A priori sample size and power calculations were conducted during the study planning stage. The calculation assumed a two-year stone recurrence rate of 40% in the placebo group and 15% in the LPR group, corresponding to a relative risk reduction of 0.25; 95%CI 0.14 – 0.44 (20). With a two-sided α of 0.05 and 80% power, the required sample size was 145 participants (72 per group). To account for an anticipated dropout rate of 10%, the target enrollment was increased to 160 participants. Full details of the power calculation are provided in the Supplementary.” In Study Design in Methods section, LINE 91-96.  - We were advised by our University’s Intellectual Property (IP) unit not to disclose the exact dosage of the main ingredient, particularly citrate, prior to patent approval, as this could potentially interfere with the patent granting process. For this reason, we have intentionally avoided emphasizing the precise amounts of citrate and potassium in the manuscript. As a compromise, we have revised the statement as follows: “*Each sachet of LPR contained 7.75 g of supplement, providing a citrate dose comparable to standard alkali therapy but with a reduced potassium content. In addition, each sachet delivered 153 mg of flavonoids, including hesperidin, diosmin, and eriocitrin. Relative to conventional potassium citrate prescriptions (30–60 mEq/day), the citrate content in a single sachet falls at the upper end of the recommended therapeutic range, whereas the potassium load remains comparatively lower*.” LINE 104-109. We wish the reviewers understand our restriction.  - The placebo had been changed to maltodextrin, as the consideration of high prevalence of lactose intolerance in elder participants. The protocol had been amended, and the detail of placebo was described in LINE 119  - We corrected the statement as mentioned above (LINE 104-109). The dose of 7.75 g LPR was determined based on the recommended daily intake of standard citrate treatment. As noted by the reviewer, we deliberately selected the upper limit of standard citrate therapy as the target dosage.  - It was our fault. 24-hour urine was collected twice. Because of this, we corrected the statement into “*The 24-hour urine samples were collected at the 0- and 24-month*”. LINE 133-134. Also, for consistency, we corrected the previous statement about blood collection into “*Blood samples were collected at 0-, 12- and 24-month*” LINE 130  - We added “*Participants who developed urolithiasis with a stone size greater than 4 mm were withdrawn from the study and referred to a urologist for appropriate management*.” LINE 141-142 |
| **Results (and Tables/Figures)**  **Line 147:** The allocation of 98 participants to the intervention arm and 75 to the placebo arm suggests an unequal randomization ratio. Could the authors clarify How was the sample size determined, and did this imbalance arise by chance due to the randomization process? Loss of participants after randomization, allocation errors, or differential withdrawal before intervention).  **Line 149**: The phrase “number of stone and recurrence rate” is confusing — “stonr number” already mentioned in the sentence and “recurrence rate” at baseline is ambiguous since recurrence is typically a follow-up outcome.  **Baseline data (Table 1):**  -Consider adding stone size as a baseline variable.  -Note that males were underrepresented compared to expected prevalence. This is noteworthy, as epidemiological evidence consistently shows that the prevalence and incidence of urolithiasis are higher in men than in women. Could it indicate selection bias in enrollment? Could the predominance of female participants influence the generalizability of the findings, given known sex-related differences in stone risk and recurrence?  Type of stone: In the Methods section, there is no description of how stone types were determined.  **Line 152**: I recommend comparing the baseline characteristics of participants who were lost to follow-up with those who completed the study. This would help assess whether attrition introduced potential bias.  **Line 166:** The section on Detection of LPR contamination appears redundant, as similar information has already been provided under LPR manufacturing, quality control and supplementation. To improve conciseness, you could consolidate these details into a single section. For example, heavy metal and pathogen testing results could be summarized once under quality control, with Table S1 referenced there if needed, rather than repeating them in multiple places.  **Line 173:** The phrase “stone recurrent rate” is not standard; the appropriate term would be “stone recurrence rate”. I recommend revising the wording accordingly.  The manuscript states that stone recurrence was monitored by KUB radiography (6 and 18 months) and CT (12 and 24 months). However, this is more a description of the imaging schedule rather than a clear definition of recurrence. For clarity, please specify: Was recurrence defined as the appearance of a new stone, the growth of a pre-existing stone, or the need for clinical intervention? Additionally, please ensure that “recurrence rate” is defined consistently in the manuscript. Clear and consistent terminology will improve readability and scientific precision.  **Line 198:** A short comment in the Discussion on the clinical relevance of decreased urinary protein and IL-8 levels in relation to stone recurrence would strengthen interpretation.  **Line 202:** Please clarify whether the reported changes in urinary protein and IL-8 are (a) within-group changes from baseline or (b) between-group differences versus placebo. For efficacy inference in an RCT, the primary analysis should compare LPR vs placebo, adjusting for baseline values. report adjusted between-group differences (or ratios if log-scaled) with 95% CIs and P-values; avoid relying on within-group significance alone.  **Line 203:** A paired t-test is appropriate for within-group baseline vs. follow-up comparisons, but not for comparing intervention and placebo groups in an RCT. For efficacy inference, the main analysis should be a between-group comparison, ideally adjusting for baseline values | - We must acknowledge that we did not fully recognize the skewed distribution of participants between the LPR and placebo groups during the study. To minimize potential bias, we engaged a third party to generate the randomization sequence using the Research Randomizer website. We were not involved in this process until the study was completed, at which point we observed the imbalance. Our best hypothesis is that the randomization sequence, by chance, led to a greater allocation to the LPR group. Alternatively, it is possible that the third party applied an unequal randomization ratio without notifying.  - We removed “, number of stone and recurrence rate” as your suggestion  - Data on stone size could not be provided due to incomplete records.  - This finding was also of interest to us. Upon further investigation through participant interviews, we found that most male participants resided in suburban areas and were required to migrate for agricultural work for approximately four months each year and subsequently worked as laborers in the city for the remainder of the year. As a result, men were less able to commit to a two-year research study. In contrast, women who remained working on the farm were more available and willing to participate. Consequently, the number of female participants exceeded that of male participants.  - We added “*The stone composition was analyzed by Fourier-transform infrared (FTIR) spectroscopy*.” In Method section LINE 126  - We added “*Table S2 Baseline demographic and clinical characteristics of participants between complete participants and drop-out*” in supplementary  - According to your suggestion, we removed this section in Result, and rewrite the Method part as “*The product was tested and certified by the Thai National Science and Technology Development Agency (NSTDA), which confirmed that heavy metals were below detection limits, which were within the acceptable limits for Daily Consumption of heavy metals - United States Pharmacopoeia (ALDC-HM USP) reference and no harmful microorganisms were present, ensuring the product's safety (Table S1).”* LINE 113-117  - We corrected the “*Stone recurrence rate*” as your suggestion LINE 186 and recheck the rest of the manuscript.  - We added “*The recurrence was diagnosed when the preexisting stone grows larger than 4 mm, or new stone was detected by radiography.*” At the Method section, LINE 128-129  - Thank you for your suggestion. We corrected the *76%* risk reduction for the abstract, figure 2 in Result, Discussion and Conclusion section, respectively.  - We added the explanation about the role of IL-18 and proteinuria with the stone disease in Discussion section as mentioned before. LINE 233-240  - As recommended, we revise the statement to “*Within-group analysis demonstrated that 24-hour urinary protein excretion and IL-8 levels were markedly reduced in participants receiving LPR compared with baseline (Figure 4). In contrast, no significant changes were observed in the placebo group. Between-group analysis showed no differences in urinary protein or IL-8 levels at baseline; however, at 24 months, both urinary protein and IL-8 levels were significantly lower in the LPR group compared with placebo (Figure S1).*” In Results section, LINE 213-217, and add the figure of comparison between-group in the supplementary Figure S1 to avoid the redundant of the figure. |
| **Discussion**  **Line 208**: The introduction and methods do not provide any information on the phytochemical content of the lime-based supplement. However, the discussion opens by emphasizing its “phytochemical-rich” properties and links the efficacy to this aspect. This could be misleading, as no phytochemical analyses were conducted or reported in the trial. The observed effects are more plausibly attributable to citrate or other components that were not specifically tested. **I recommend revising the discussion to focus on conclusions that can be directly supported by the RCT data (e.g., changes in recurrence, urinary parameters, safety outcomes),** while avoiding speculative statements about phytochemicals unless supported by measured evidence. If phytochemicals are to be discussed, they should be presented clearly as a potential mechanism requiring further study rather than as a proven explanation.  **Line 210:** Please discuss **cost-effectiveness** of LPR compared to standard potassium citrate therapy. Cost-effectiveness is a key factor in long-term adherence and clinical adoption, particularly in low-resource settings where stone recurrence burden is high. Addressing whether LPR offers an economic advantage compared with existing pharmacological options would provide valuable context for interpreting its potential role in clinical practice.  **Line 225**: Here In the discussion, you state that urinary protein reflects both glomerular and tubular damage associated with crystalluria and inflammation. However, this important claim is currently supported by only one small, self-cited study. For a statement of this scope, I strongly recommend citing multiple, higher-quality sources. In particular, please incorporate recent reviews and large observational studies that: Document crystal-induced tubular injury and inflammatory pathways in kidney stone disease. Summarize evidence for tubular injury biomarkers (e.g., KIM-1, NGAL) and their relevance to stone pathophysiology. Highlight epidemiologic data linking albuminuria/proteinuria with increased risk of nephrolithiasis. Expanding the reference base in this way will strengthen the scientific foundation of your argument and avoid overreliance on a single study from your own group.  **Line 230**: Flavonoids and related polyphenols can reduce oxidative stress/inflammation, modulate crystal growth/adhesion, and influence crystal–cell interactions in vitro/animal models; clinical translation remains limited. If mentioned, please frame as hypothesis and cite reviews rather than implying causality. While citrus-derived phytochemicals have been shown in preclinical models to reduce oxidative stress, modulate crystal growth/adhesion, and attenuate inflammatory signaling, including pathways related to IL-8, these mechanisms were not directly evaluated in our trial. The observed clinical benefit is more parsimoniously explained by alkali/citrate effects, consistent with prior citrus-juice and citrate literature; phytochemical mechanisms should be viewed as hypotheses for future targeted studies.  **Line 251**: In the conclusion, the manuscript emphasizes “phytochemical-based therapies” as a potential strategy for secondary prevention. However, this conclusion is not supported by the data presented. The trial did not measure or report the phytochemical content of the intervention, nor did it test mechanistic pathways to link phytochemicals with outcomes. The observed benefits are more directly attributable to the citrate content of the supplement and related metabolic effects.  **Line 260**: It is a valuable consideration that dietary intake was assessed across groups, as diet can influence urinary chemistry and stone recurrence. I suggest repeated dietary recalls for this matter. | - We added “*and 153 mg of flavonoid, including hesperidin, diosmin and eriocitrin*.” in Method section, LINE 106.  - As your recommendation, we revise the whole Discussion section, emphasize the changes in recurrence, urinary parameters, safety outcomes, followed by the hypothesis of mechanism of action and. Please be noted that even we indicated the restriction for using LPR in calcium phosphate stone, however, another group is currently investigating the LPR in treatment of kidney stone at Nan province, Thailand and found that LPR effectively reduce calcium oxalate and calcium phosphate, regardless urine pH alkalizing effects.  - We appreciate the reviewers’ interest in comparing the cost-effectiveness of LPR with standard therapy. However, a direct comparison is difficult at this stage, as LPR has not yet been commercially manufactured. Based on the production costs from a GMP-certified manufacturer used in this study, the estimated cost of LPR was approximately 15 Baht per day (approximately 0.47 USD). For reference, the procurement cost of potassium citrate for public hospitals in Thailand (not the retail price to patients, which varies) is also about 15 Baht per day. We anticipate that the production cost of LPR could be substantially reduced with large-scale commercial manufacturing. At this stage we can only add “*Additionally, the estimated cost of LPR is comparable to, or potentially lower than, that of standard potassium citrate therapy.*” In Discussion section, LINE 270-271  - We have revised the references as follows: Ref. 22 now cites “*Management of Urinary Stones by Experts in Stone Disease (ESD 2025)*,” which is a review article and practice guideline (Lines 228–229). In addition, Ref. 20 has been updated to cite a review article entitled “*Interleukins in Urological Diseases*,” which discusses the role of IL-18 in urolithiasis, LINE 233-240.  We hope that this revision fulfils your suggestion.  - We really appreciate your advice. WE revised our statement as “*Although these phytochemicals have not yet been proven effective in the treatment of stone disease in humans, they represent promising nutraceutical candidates for stone prevention. Further clinical studies are required to confirm their efficacy. Taken together, citrate-mediated urine alkalinization, in combination with antioxidant activity, offers a multifaceted mechanism for the prevention of recurrent stones.”* In Discussion section, LINE 261-265  - We apologize for omitting the flavonoid data in the previous version. We have now added this information in the Methods section, LINE 104-109 as follows: “*In addition, each sachet delivered 153 mg of flavonoids, including hesperidin, diosmin, and eriocitrin*.” However, we are unable to disclose the exact amounts of individual flavonoids (hesperidin 26.64 mg, diosmin 3.32 mg, and eriocitrin 1.54 mg) due to restrictions from our University’s Intellectual Property (IP) unit, as mentioned previously.  - We greatly appreciate this valuable suggestion and fully agree with the reviewer. In future studies, we plan to repeat dietary recalls with a larger number of participants to strengthen the analysis. |
| **Conclusion**  •I recommend revising the conclusion to reflect findings that are directly supported by this RCT—namely, the observed reduction in stone recurrence and urinary/biochemical changes with LPR. Phytochemicals may be mentioned only as a hypothesis-generating area for future research, rather than as evidence-based therapies suitable for guideline integration at this stage.  •The reported 75.6% reduction in recurrence should be clarified as relative vs absolute risk, and effect sizes presented with confidence intervals.  • Statements about uric acid and calcium phosphate stones are speculative. Please restrict conclusions to calcium-based stones, which were studied.  o The trial enrolled patients with calcium-based stones (predominantly calcium oxalate). Therefore, claims that LPR may also benefit patients with uric acid stones or be ineffective for calcium phosphate stones are speculative and not supported by your data. I recommend revising the conclusion to clearly state that the findings apply to calcium-based stone formers only, and note that efficacy in other stone types requires further investigation.  • The mechanisms listed (citrat­uria, alkalinization, antioxidant/anti-inflammatory effects from citrus flavonoids) should be revised. None of these were directly demonstrated in this trial. The proposed role of flavonoids is speculative, as no phytochemical analyses or mechanistic assessments were conducted. Please frame flavonoid-related mechanisms as hypotheses for future research rather than established findings. While it is appropriate to discuss these possibilities in the Discussion, they should not be included in the Conclusion. Revise to highlight evidence-based findings: reduced recurrence and improved urinary parameters in calcium-based stone formers.  • Emphasize that safety was observed over 2 years, but long-term tolerability remains to be evaluated. Maintain caution regarding potassium use in advanced CKD, with reference to guidelines. | - We revised the conclusion as your suggestion “*This study provides strong clinical evidence that LPR supplementation significantly decreases the risk of recurrent calcium oxalate stones. Over 24 months, participants receiving LPR experienced a 76% relative risk reduction in recurrence, with absolute risk reduction 35.7%, in recurrence compared with placebo, accompanied by improvements in urinary biomarkers of renal injury, including reductions in protein excretion and IL-8 levels. LPR was well tolerated, with no hepatic or renal toxicity observed, supporting its safety for long-term use.*  *The clinical benefits observed in this study likely reflect attenuation of renal inflammation, while previous studies from our group have demonstrated that LPR also increases urinary citrate excretion and urine pH, supporting its mechanistic plausibility. While the flavonoid content of LPR may contribute to these protective effects, its role remains hypothesis-generating and requires further validation in human studies.*  *Taken together, our findings support LPR as a safe, culturally acceptable, and cost-effective intervention for secondary prevention of calcium oxalate urolithiasis, with potential for integration into clinical management strategies pending confirmation in larger, longitudinal trials*.” In Conclusion section  - We revised “*The hazard ratio for recurrence in the LPR group compared to the placebo group was 0.24 (95% CI: 0.13–0.44; p < 0.0001), relative risk reduction was 76% and absolute risk reduction was 35.7%”* in Results section LINE193-195 and added “Over 24 months, participants receiving LPR experienced a 76% relative risk reduction in recurrence, with absolute risk reduction 35.7%,” in Conclusion section, LINE 294-295  *-* We removed “*In contrast, LPR appears particularly effective for preventing recurrence in patients with calcium oxalate or uric acid stones.*” From Discussion section as your suggestion  - We have revised the Conclusion section according to your suggestion. In line with your concern, we also removed mention of other types of stone treatment, as this content did not align with the title or the primary objective of the study.  - We revised the statement to “*The clinical benefits observed in this study likely reflect attenuation of renal inflammation, while previous studies from our group have demonstrated that LPR also increases urinary citrate excretion and urine pH, supporting its mechanistic plausibility*.” As your suggestion LINE 302-304  - We revised “*LPR was well tolerated over the 2-year trial period, with no hepatic or renal toxicity observed; however, long-term tolerability beyond this timeframe remains to be established. In accordance with current guideline recommendations, caution should be exercised in patients with advanced chronic kidney disease, particularly due to the potassium content of the supplement*.” In Conclusion section as your suggestion. LINE 297-301 |
| **Comments to the author checklist** | We added more statistical analysis as the reviewers’ suggestion and revised the Statistical Analysis in Method section to be more cleared, as followed “*All randomized participants were included in the analysis. For the primary outcome, time to stone recurrence was evaluated using a Cox proportional hazards model to estimate hazard ratios (HRs) with 95% confidence intervals (CIs). Kaplan–Meier curves were constructed to illustrate stone-free survival.*  *For continuous variables, data distribution was first assessed using the Shapiro–Wilk test. Parametric data are presented as means ± standard deviations, and non-parametric data as medians with interquartile ranges. Between-group comparisons were performed using independent-sample t-tests or Mann–Whitney U tests, as appropriate. Within-group comparisons were conducted using paired t-tests or Wilcoxon signed-rank tests.*  *All statistical tests were two-sided, and a p-value < 0.05 was considered statistically significant. Data visualization was performed using bar graphs with error bars representing standard deviations or interquartile ranges. Analyses were conducted using SPSS software, version 21.0 (SPSS Inc., Chicago, IL, USA) and GraphPad Prism version 10.1.1 for macOS (GraphPad Software, Boston, MA, USA).”* In Methods section, LINE 145-156 |
| **Reviewer 2**  1)I could not see any power calculations in the main paper, please include at least a mention and that fully calculations are in the protocol.  2) it is supposed to be 1:1 but I see more people in the intervention. This does not seem to be linked to different drop out rates in the 2 groups. clarification is needed.  3) why wasn't the randomisation conducted within each centre for better balance?  4) A Cox model is mentioned in the methods section, with no covariates listed for adjustment - then KM curves are presented (which are fine if there is perfect balance across the covariates of interest - in this case there is no perfect balance on location despite this being listed as NS, since p-values are a function of sample size and in this case the sample size is low). So to summarise, A Cox model with location as a covariate would probably run and adjust for potential differences in diets that are location driven. if randomisation was done within stratum, this would not be an issue. If there is geographic variation in stone risk factors in Thailand, failing to adjust for location may introduce bias.  5) the analyses do not account for location. ideally this would be done using a random effect (shared frailty) in a Cox regression model, if the numbers allow.  6) The use of randomizer.org is mentioned, but more details are needed e.g. on allocation concealment and sequence generation etc  7) There are many secondary outcomes and the findings from these analyses should be explored with caution, considering the small sample size and the fact no corrections for multiple testing were applied. any findings need to be clearly be stated as exploratory and this needs to be discussed as a limitation in the relevant section.  8) The paper states that 151 out of 173 participants completed the study, but it’s unclear how dropouts were handled in the analysis. Clarify ITT was used, an important point since dropout rates were non-negligible.  **Minor**  1) report exact p-values to 3 decimals, don't just state NS  2) Some figures lack confidence intervals or number-at-risk tables, which are standard for survival analysis reporting.  3) The checklist marks several items as NA which contradicts the manuscript content.  4) The manuscript states no missing data for primary outcomes, but it’s unclear how missing data for secondary outcomes were handled.  5) If there is a taste difference between LPR and placebo, more detail on how blinding was assessed or maintained would be helpful.  6) A table summarising all secondary outcomes with effect sizes and confidence intervals would help readers interpret the findings more cautiously. | - We demonstrated the sample size calculation as additional data in Supplementary. As your suggestion, we added “*A priori sample size and power calculations were conducted during the study planning stage. The calculation assumed a two-year stone recurrence rate of 40% in the placebo group and 15% in the LPR group, corresponding to a relative risk reduction of 0.25; 95%CI 0.14 – 0.44 (20). With a two-sided α of 0.05 and 80% power, the required sample size was 145 participants (72 per group). To account for an anticipated dropout rate of 10%, the target enrollment was increased to 160 participants. Full details of the power calculation are provided in the Supplementary*.” In the Study Design of Methods section, LINE 91-96  - We described the unexpected discrepancy between participant numbers of each group previously. To minimize potential bias, we engaged a third party to generate the randomization sequence using the Research Randomizer website. We were not involved in this process until the study was completed, at which point we observed the imbalance. Our best hypothesis is that the randomization sequence, by chance, led to a greater allocation to the LPR group. Alternatively, it is possible that the third party applied an unequal randomization ratio without notifying.  - For 4) and 5) We appreciate the reviewer’s insightful comment regarding the potential influence of location on stone risk factors. In this trial, randomization was performed centrally without stratification by location. As a result, the number of participants enrolled across study regions was somewhat unbalanced. Although baseline characteristics were not statistically different between groups, we acknowledge that p-values may be underpowered to detect true imbalance, particularly given the modest sample size. Unfortunately, stratified randomization was not pre-specified, and reanalysis adjusting for location was not included in the original statistical plan. We agree that geographic variation could represent a potential source of residual confounding, and we have added this point, together with the issue of unequal participant distribution across regions, to the limitations section of the revised manuscript.  “*randomization was not stratified by study location, leading to some imbalance in participant numbers across regions. Although baseline characteristics were comparable, the modest sample size may have limited our ability to detect subtle geographic differences*.” In Discussion section, LINE 289-291  - We regret that further details on the randomization process cannot be provided. As previously noted, randomization was conducted by a third party, and the detailed records are no longer available because they have since been destroyed.  - We appreciate your suggestion, which is consistent with the comments of other reviewers. In the revised Discussion, we have focused on stone recurrence, urinary parameters, and safety outcomes, while noting that the potential benefits of flavonoids require further investigation. We hope this revision addresses your concern.  - We added “*Participants who developed urolithiasis with a stone size greater than 4 mm were withdrawn from the study and referred to a urologist for appropriate management*.” LINE 141-142  - We added the p-value of each variable in Table 1 as your suggestion.  - We added the number-at-risk tables in Figure 2, and revised the legend of figure to “*Kaplan–Meier survival analysis demonstrated the time to stone recurrence in the LPR and placebo groups over a 24-month follow-up period. Numbers at risk are displayed below the x-axis. The LPR group exhibited a significantly lower risk of recurrence compared with the placebo group, corresponding to an approximate 76% relative risk reduction (hazard ratio [HR] = 0.24; 95% CI: 0.13–0.44; p < 0.0001).”* in Figure 2 LINE 198-202  - We appreciate your concern. We will correct this during the re-submission.  - We thank the reviewer for this valuable comment. We confirm that there was no missing data for either the primary or secondary outcomes. All participants were required to attend hospital visits at each follow-up for ultrasound or CT scans, and during the same visits, urine and blood samples were consistently collected. This ensured complete data capture for both recurrence and biochemical parameters. We have revised the Methods section to clarify this point. “*All participants attended scheduled hospital visits every six months for imaging assessments (ultrasound or CT scan). During these visits, urine and blood samples were collected, ensuring complete data captured for both primary and secondary outcomes*.” In Method section, LINE 121-124  The higher dropout rate at the first follow-up was mainly due to participants being referred for stone treatment at the central hospital. Although they initially consented to join the study, subsequent follow-up at our research center was not possible because their healthcare coverage did not allow continued referral.  - We appreciate the reviewer’s concern. As the taste of LPR and maltodextrin differs, complete masking was not feasible. To minimize bias, participants were not informed at enrollment that the investigational product was lime-based, and they were only told that the supplement might taste mildly sweet or sour.  - We added the statement of “*Summarization of secondary outcomes was demonstrated in Table S4.”* In Results section, LINE 223, and Table S4 Summarization of secondary outcomes and interpretation in Supplementary |
| **Reviewer 3**  1. why were patients with more than one stone excluded from the study?  were all patients with stones asymptomatic?  2.were the stones analyzed after lithotripsy, how do we know that they were all mainly of calcium oxalate stones? HU could help as proxy in that matter. Were HU measured for each stone and what was the threshold for considering a stone of calcium oxalate?  3.non contrast CT is the imaging of choice for detection of stones before and after lithotripsy  US and KUB are far less reliable especially for smaller stones, please explain why CT was not used for all patients consistently especially given that stone free rates after surgery cannot be reliably evaluated with US  4. how many patients had lithotripsy before study enrollement?  5.what is the amount of citrates delivered by LPR? compared to the suggested minimmum daily dietary intake? this information is missing  6.why ALT in particular was measured?  7. table 1: how is this percentage of calcium oxalate stones estimated since no relevant studies were done (stone analysis or use of HU?  8. discussion: discuss the clinical implications of elevated urine protein and IL8  9. discussion:what is the correlation between renal tissue injury and calcium stone formation? | - We apologize for the misunderstanding. All participants, including those with multiple stones, were enrolled in the study  - All stones were retrieved and analyzed. We added “*The stone composition was analyzed by Fourier-transform infrared (FTIR) spectroscopy*.” In Method section LINE 126  - We appreciate the reviewer’s comment. While CT scanning is recognized as the gold standard for detecting stones, its availability is limited in our setting. Therefore, post-stone removal ultrasound was considered an acceptable alternative in our country.  - All participants had performed lithotripsy, either extracorporeal shockwave, or laser lithotripsy.  - The citrate content in LPR was approximately 65 mEq/day in this study. However, we were advised by our University’s Intellectual Property (IP) unit not to disclose the exact dosage of the main ingredient, particularly citrate, prior to patent approval, as this could potentially interfere with the patenting process. For this reason, the exact value was not reported in the manuscript. As a compromise, we have revised the statement as follows: “*Each sachet of LPR contained 7.75 g of supplement, providing a citrate dose comparable to standard alkali therapy but with a reduced potassium content*.” LINE 104-105  - All stones were analyzed by FTIR  - We added “*The observed clinical benefits are supported by mechanistic plausibility. Crystal-induced epithelial injury and micro-obstruction in stone disease promote local inflammation and cytokine release, including IL-8, thereby sustaining oxidative stress and facilitating crystal retention. Elevated urinary protein similarly reflects obstruction- or injury-related disruption of glomerular and tubular integrity. Reductions in IL-8 and proteinuria following LPR supplementation suggest attenuation of renal inflammation and restoration of barrier function, consistent with recovery of renal health (21). Prior recommendations from the Experts in Stone Disease (ESD) Conference also emphasize the utility of proteinuria monitoring in high-risk stone formers, underscoring the clinical relevance of these markers (22)*” in Discussion section, LINE 233-240  - We added “*Renal tissue injury is closely linked to calcium oxalate stone formation through a self-reinforcing cycle. Exposure to oxalate and calcium oxalate crystals induces reactive oxygen species, localized inflammation, and tubular injury (15). In addition, injury to the renal tubular or papillary epithelium exposes underlying matrix components that provide adhesive sites for calcium oxalate crystal attachment. Once crystals adhere, they trigger oxidative stress and local inflammation, particularly through cytokines such as IL-6 and IL-8, which amplify tubular damage. Interstitial calcium phosphate deposits (Randall’s plaques) exposed to urine can also act as a nidus for calcium oxalate overgrowth as well as activation of NLRP3 inflammasome, leading to maturation and release of IL-1β and IL-18 pro-inflammatory cytokines and cell death (16). As calcium oxalate stones enlarge or pass, they cause further epithelial trauma and obstruction, perpetuating injury and increasing the risk of recurrence. Thus, renal tissue injury not only facilitates calcium oxalate stone initiation but is also exacerbated by stone growth and passage*.” In Discussion section, LINE 241-251 |
