# Supplementary material for: Lime-based supplement reduces calcium oxalate stone recurrence: A multicenter randomized controlled trial
Source: PLoS One. 2025 Dec 5;20(12):e0336892. doi: 10.1371/journal.pone.0336892 (PMC12680185; doi:10.1371/journal.pone.0336892)
Supplement: Attachment — Submitted filename: Reviewers comments 2.docx [file pone.0336892.s006.docx]

Response to Reviewers #2

| Reviewer’s recommendation | Correction |
| --- | --- |
| Reviewer #1  - In the paragraph beginning with “It is important to note certain limitations”, it would be more appropriate to change the term “limitations” to “considerations”.  - the statement “our previous studies have consistently shown that LPR supplementation modulates urinary metabolic risk factors, supporting the biological plausibility of our findings” requires an appropriate citation to substantiate this claim.  - The explanation provided for the gender imbalance — that male participants were less available due to seasonal migration for work — is informative and contextually relevant. However, this should also be acknowledged as a limitation, since the overrepresentation of female participants may affect the generalizability of the study’s findings to broader populations, particularly to males who may differ in metabolic or lifestyle characteristics influencing stone risk or treatment response. | - Thank you for your suggestion. We corrected the statement into “It is important to note certain considerations.” Page 16, Line 266, yellow highlighted  - Thank you for your suggestion. We added the citation (8, 19), which were our studied about the urinary metabolic profile after LPR supplementation into the statement, Page 16, Line 282, yellow highlighted  - We added this into the 5^th^ consideration as “Fifth, the predominance of female participants in this study, despite the higher prevalence of urolithiasis among males, represents a limitation. Because many male patients were unable to participate due to frequent work-related relocations, this sex imbalance may limit the generalizability of our findings to the broader population.” Page 17, Line 290-293, yellow highlighted |

| Reviewer’s recommendation | Correction |
| --- | --- |
| Reviewer #2: I struggled to follow the responses. can the authors provide clear, point by point responses for each specific question and comment? | - We apologize for the reviewer’s inconvenience. Following is the previous Response to reviewers #1, point-by-point in reorganized pattern, with blue highlight in the manuscript |

*Response to reviewers #1*

| **Reviewer 2**  1)I could not see any power calculations in the main paper, please include at least a mention and that fully calculations are in the protocol. | - We demonstrated the sample size calculation as additional data in Supplementary. As your suggestion, we added “*A priori sample size and power calculations were conducted during the study planning stage. The calculation assumed a two-year stone recurrence rate of 40% in the placebo group and 15% in the LPR group, corresponding to a relative risk reduction of 0.25; 95%CI 0.14 – 0.44 (20). With a two-sided α of 0.05 and 80% power, the required sample size was 145 participants (72 per group). To account for an anticipated dropout rate of 10%, the target enrollment was increased to 160 participants. Full details of the power calculation are provided in the Supplementary*.” In the Study Design of Methods section, LINE 91-96 |
| --- | --- |
| 2) it is supposed to be 1:1 but I see more people in the intervention. This does not seem to be linked to different drop out rates in the 2 groups. clarification is needed.  3) why wasn't the randomisation conducted within each centre for better balance? | - We must acknowledge that we did not fully recognize the skewed distribution of participants between the LPR and placebo groups during the study. To minimize potential bias, we engaged a third party to generate the randomization sequence using the Research Randomizer website. We were not involved in this process until the study was completed, at which point we observed the imbalance. Our best hypothesis is that the randomization sequence, by chance, led to a greater allocation to the LPR group. Alternatively, it is possible that the third party applied an unequal randomization ratio without notifying. |
| 4) A Cox model is mentioned in the methods section, with no covariates listed for adjustment - then KM curves are presented (which are fine if there is perfect balance across the covariates of interest - in this case there is no perfect balance on location despite this being listed as NS, since p-values are a function of sample size and in this case the sample size is low). So to summarise, A Cox model with location as a covariate would probably run and adjust for potential differences in diets that are location driven. if randomisation was done within stratum, this would not be an issue. If there is geographic variation in stone risk factors in Thailand, failing to adjust for location may introduce bias.  5) the analyses do not account for location. ideally this would be done using a random effect (shared frailty) in a Cox regression model, if the numbers allow. | - For 4) and 5) We appreciate the reviewer’s insightful comment regarding the potential influence of location on stone risk factors. In this trial, randomization was performed centrally without stratification by location. As a result, the number of participants enrolled across study regions was somewhat unbalanced. Although baseline characteristics were not statistically different between groups, we acknowledge that p-values may be underpowered to detect true imbalance, particularly given the modest sample size. Unfortunately, stratified randomization was not pre-specified, and reanalysis adjusting for location was not included in the original statistical plan. We agree that geographic variation could represent a potential source of residual confounding, and we have added this point, together with the issue of unequal participant distribution across regions, to the limitations section of the revised manuscript.  “*randomization was not stratified by study location, leading to some imbalance in participant numbers across regions. Although baseline characteristics were comparable, the modest sample size may have limited our ability to detect subtle geographic differences*.” In Discussion section, LINE 293-296 (blue highlighted) |
| 6) The use of randomizer.org is mentioned, but more details are needed e.g. on allocation concealment and sequence generation etc | - We regret that further details on the randomization process cannot be provided. As previously noted, randomization was conducted by a third party, and the detailed records are no longer available because they have since been destroyed. |
| 7) There are many secondary outcomes and the findings from these analyses should be explored with caution, considering the small sample size and the fact no corrections for multiple testing were applied. any findings need to be clearly be stated as exploratory and this needs to be discussed as a limitation in the relevant section. | - We appreciate your suggestion, which is consistent with the comments of other reviewers. In the revised Discussion section, we have focused on stone recurrence, urinary parameters, and safety outcomes, while noting that the potential benefits of flavonoids require further investigation. We hope this revision addresses your concern. LINE 225-296, no highlighted |
| 8) The paper states that 151 out of 173 participants completed the study, but it’s unclear how dropouts were handled in the analysis. Clarify ITT was used, an important point since dropout rates were non-negligible. | - We added “*Participants who developed urolithiasis with a stone size greater than 4 mm were withdrawn from the study and referred to a urologist for appropriate management*.” LINE 141-142  The higher dropout rate at the first follow-up was mainly due to participants being referred for stone treatment at the central hospital. Although they initially consented to join the study, subsequent follow-up at our research center was not possible because their healthcare coverage did not allow continued referral. |
| **Minor**  1) report exact p-values to 3 decimals, don't just state NS | - We added the p-value of each variable in Table 1 as your suggestion. |
| 2) Some figures lack confidence intervals or number-at-risk tables, which are standard for survival analysis reporting. | - We added the number-at-risk tables in Figure 2 and revised the legend of figure to “*Kaplan–Meier survival analysis demonstrated the time to stone recurrence in the LPR and placebo groups over a 24-month follow-up period. Numbers at risk are displayed below the x-axis. The LPR group exhibited a significantly lower risk of recurrence compared with the placebo group, corresponding to an approximate 76% relative risk reduction (hazard ratio [HR] = 0.24; 95% CI: 0.13–0.44; p < 0.0001).”* in Figure 2 LINE 198-202, blue highlighted |
| 3) The checklist marks several items as NA which contradicts the manuscript content. | - We appreciate your concern. We will correct this during the re-submission. |
| 4) The manuscript states no missing data for primary outcomes, but it’s unclear how missing data for secondary outcomes were handled | - We thank the reviewer for this valuable comment. We confirm that there was no missing data for either the primary or secondary outcomes. All participants were required to attend hospital visits at each follow-up for ultrasound or CT scans, and during the same visits, urine and blood samples were consistently collected. This ensured complete data capture for both recurrence and biochemical parameters. We have revised the Methods section to clarify this point. “*All participants attended scheduled hospital visits every six months for imaging assessments (ultrasound or CT scan). During these visits, urine and blood samples were collected, ensuring complete data captured for both primary and secondary outcomes*.” In Method section, LINE 121-124 |
| 5) If there is a taste difference between LPR and placebo, more detail on how blinding was assessed or maintained would be helpful. | - We appreciate the reviewer’s concern. As the taste of LPR and maltodextrin differs, complete masking was not feasible. To minimize bias, participants were not informed at enrollment that the investigational product was lime-based, and they were only told that the supplement might taste mildly sweet or sour. |
| 6) A table summarising all secondary outcomes with effect sizes and confidence intervals would help readers interpret the findings more cautiously. | - We added the statement of “*Summarization of secondary outcomes was demonstrated in Table S4.”* In Results section, LINE 223, and Table S4 Summarization of secondary outcomes and interpretation in Supplementary |
